# Renal effects of a novel endogenous natriuretic agent xanthurenic acid 8-*o*-*β*-d-glucoside in rats

**DOI:** 10.1002/phy2.155

**Published:** 2013-11-13

**Authors:** Aaron Hoffman, Marina Okun-Gurevich, Elena Ovcharenko, Ilia Goltsman, Tony Karram, Cristopher Cain, Zaid Abassi, Joseph Winaver

**Affiliations:** 1 Department of Vascular Surgery and Transplantation, TechnionHaifa, Israel; 2 Department of Physiology and Biophysics, Faculty of Medicine, Rambam Medical Center and the Rappaport Research Institute, TechnionHaifa, Israel; 3 Naturon Pharmaceutical CorporationNew Canaan, Connecticut

**Keywords:** Amiloride-sensitive sodium channel, kidney, kynurenine pathway, natriuretic hormone, nitric oxide, rat

## Abstract

Xanthurenic acid 8-*o-β*-d-glucoside is an endogenous derivative of tryptophan metabolism, isolated from urine of patients with chronic renal disease. This compound was suggested previously to act as a natriuretic hormone based on its ability to block short circuit currents in a frog skin assay and to induce a sustained natriuresis when injected into rats (C. D. Cain et al., *Proc. Natl. Acad. Sci. USA* 2007: 17873–17878). The present communication describes the effects of the compound on renal clearance and hemodynamic parameters in male Sprague–Dawley rats maintained on a normal salt (0.4–0.5%) diet. Intravenous administration of synthetic xanthurenic acid 8-*o-β*-d-glucoside in two consecutive incremental doses (6.3 and 31.5 nmol) resulted in a significant increase (*P* < 0.05), in urine flow (43.91 ± 6.31 *μ*L/min vs. 10.54 ± 2.21 *μ*L/min), absolute rate of sodium excretion (3.99 ± 0.95 *μ*Eq/min vs. 1.15 ± *μ*Eq/min), and percentage sodium excretion (1.63 ± 0.46% vs. 0.37 ± 0.12%, peak response vs. baseline, respectively). The natriuretic/diuretic effect was associated also with a significant increase in potassium excretion. These effects were not related to changes in renal hemodynamics or in arterial blood pressure. Pretreatment with the sodium channel blocker, amiloride, completely abolished the natriuretic and kaluretic actions of the compound. Administration of the xanthurenic acid derivative caused a dose-related increase in urinary nitrite/nitrate excretion. Moreover, under chronic nitric oxide blockade by l-NG-Nitro-Arginine-Methyl-Esther (l-NAME) sodium excretion was similar in rats treated or untreated with the compound. Our data demonstrate that xanthurenic acid 8-*o-β*-d-glucoside has significant diuretic/natriuretic and kaluretic properties. An intact amiloride-sensitive sodium channel is required for the renal effects of the compound. The data further suggest that the natriuretic effect is mediated in part by a nitric oxide-dependent mechanism.

## Introduction

The notion that endogenous agents acting to promote sodium excretion by the kidney is released in response to salt loading and extracellular fluid volume expansion, was originally proposed in the early 1960s by De Wardener et al. (1961).

Since then, numerous in vivo and in vitro studies provided additional evidence to the existence of such “natriuretic hormones” (for reviews see Bricker et al. 1975; de Wardener and Clarkson 1985; Buckalew and Paschal-McCormick 1989). Indeed, several studies reported the natriuretic activity of low-molecular-weight (<1000 Da) substances present in the plasma and urine of salt- and volume-loaded humans (Clarkson et al. 1976; Epstein et al. 1978) as well as in patients with chronic renal disease (Bourgoignie et al. 1974; Bricker et al. 1993). However, attempts to identify the nature of the natriuretic substance(s) in extracts of plasma and urine were unsuccessful.

Cain et al. (2007) identified a new class of human natriuretic substances in the urine of patients with chronic renal disease. These molecules are derivatives of xanthurenic acid: namely, the 8-*o-β*-d-glucoside and 8-*o*-sulfate. The two compounds were partially purified by fractionation and subsequently isolated by nuclear magnetic resonance (NMR) spectroscopy of the enriched fractions. The active fractions were shown to decrease sodium transport, measured as short circuit currents in a frog skin assay, and to induce sustained natriuresis when injected to rats (Cain et al. 2007).

Xanthurenic acid is a metabolic product of the essential amino acid tryptophan, via the kynurenine pathway (Schwarcz and Pellicciari 2002; Stone and Darlington 2002). In the brain, tryptophan and its metabolites act as important neurotransmitters; however, xanthurenic acid and other tryptophan derivatives are found also in other organs, including liver and kidney (Schwarcz and Pellicciari 2002; Allegri et al. 2003).

The purpose of this study was to evaluate the effects of the 8-*o-β*-d-glucoside derivative of xanthurenic acid (XAG) on renal electrolyte and water excretion and renal hemodynamics, using clearance methodology and direct measurement of renal blood flow (RBF), and to assess the possible mechanisms of action of the compound. In particular, we evaluated whether the amiloride-sensitive sodium channel in the collecting duct and the nitric oxide (NO) system are involved in the mechanism of the natriuretic action of XAG.

## Material and Methods

Studies were performed in male Sprague-Dawley (SD) rats (Harlan Laboratories, Jerusalem, Israel), weighing 280–320 g (10–11 weeks old) that were housed in individual cages and maintained on standard commercial rat chow (containing 0.4–0.5% NaCl) and tap water ad libitum. Experiments were performed according to the Guides for the Care and Use of Laboratory Animals (NIH Publication No. 85-23, revised 1996), as approved by the local Institutional Committee for Animal Experiments.

Synthetic xanthurenic acid 8-*o-β*-d-glucoside was kindly supplied by Dr. N.S. Bricker and Dr. M. Mitchnick (Naturon and Particle Sciences, Inc, Bethlehem, PA).

## Experimental protocols

### Clearance studies

#### Effects of XAG on mean arterial pressure, glomerular filtration, and renal excretory parameters in normal rats

On the day of the experiment, rats were anesthetized by injection of inactin (sodium thiobutabarbital, 100 mg/kg BW, i.p.; Sigma-Aldrich Co., St Louis, MO), placed on a thermoregulated operating table and prepared for clearance studies as described previously (Winaver et al. 1988). Following tracheostomy, PE-50 catheters were inserted into the left carotid artery, jugular vein, and the urinary bladder for continuous BP monitoring, blood sampling, infusion of various solutions, and urine collection, respectively. Mean arterial pressure (MAP) was continuously measured by a pressure transducer (model 1050.1; UFI, Morro Bay, CA) through the carotid arterial line and recorded (B.P instrument, model 50110; Stoelting Co., Wood Dale, IL). A 0.9% saline solution containing 2.0% inulin was continuously infused at a rate of 1.0–1.5% of BW per hour throughout the experiment. Fluid infusion has been adjusted in all experiments to account for volume loss. After two baseline clearance periods of 30 min each, XAG dissolved in 0.5 mL of 0.9% saline was administered intravenously as a slow infusion over a 10-min period, in two consecutive incremental doses of 6.3 and 31.5 nmol, (based on Cain et al. 2007) (low and high dose, respectively). Each dose was followed by two additional 30-min clearance periods.

Blood samples (0.3 mL) were obtained every second clearance period, separated by centrifugation, and analyzed for inulin and electrolytes. Urine volume was measured gravimetrically. Glomerular filtration rate (GFR) was equated with the clearance of inulin (C_in_).

#### Effects of blockade of the amiloride-sensitive sodium channel on renal actions of XAG

Normal rats were prepared for clearance studies as described in the early section with the exception that amiloride was dissolved in the inulin solution and the concentration was adjusted to deliver a priming dose of 5.0 mg/kg, administered over a period of 10 min, followed by a sustained infusion 10.0 mg/kg × hour throughout the entire experiment. The rats were then randomly divided into two groups. In the first (*N* = 7), XAG was injected after baseline collections in identical doses as described in the previous protocol. A second group (*N* = 9) served as controls and were injected with vehicle (saline) instead of XAG. Urine collections and blood samples were obtained as described previously.

#### Role of the NO system in the renal actions of XAG

To evaluate whether the renal NO system is involved in the mediation of the renal actions of XAG 2 experimental protocols were utilized. In the first protocol, urine samples were obtained for measurements of urinary nitrite/nitrate in normal rats that were either treated with two incremental doses of XAG in an identical protocol as described in protocol A (*N* = 6) or vehicle (saline) only (*N* = 5). In these experiments, urine was collected under ice and samples were kept frozen (−70°C) until assayed. In the second protocol, normal rats were treated by adding l-NAME to drinking water (50.0 mg/L) for 4 days prior to the clearance experiment. The animals were then randomly divided into two groups; the first (*N* = 10) was subjected to administration of XAG according to protocol A. The second group (*N* = 10) was injected with vehicle (saline) and served as a time control group. Urine and plasma samples were obtained and handled as described in protocol A.

##### Renal hemodynamics measurements

For renal hemodynamic measurements, rats were anesthetized and similarly catheterized as described in the previous protocol. The left kidney was then exposed through a mid-abdominal incision and an ultrasonic probe (IRB type; Transonic systems, Inc., Ithaca, NY) was placed around the renal artery. Total RBF was measured by an ultrasonic flowmeter (model T206; Transonic systems), as previously described from our laboratory (Brodsky et al. 1998). MAP was continuously recorded by a pressure transducer connected to the arterial line as described in the previous protocol.

Following surgery and stabilization phase, recordings of MAP and RBF were started. Averaged 60-sec baseline recordings of MAP and RBF were obtained every 5 min for a baseline period of 30 min. Then, XAG in two consecutive incremental doses was administered i.v. over a 10-min period, as described in the previous protocol. Each dose of the compound was followed by additional MAP and RBF recordings of 30–40 min, as described for the baseline period. Renal vascular resistance (RVR) was calculated online by a computerized data acquisition system (Labtech Notebook®; Labtech, Andover, MA) as described previously (Brodsky et al. 1998).

### Analytical methods

Concentrations of sodium and potassium in urine and plasma samples were determined by a flame photometer (model 943; Instrumentation Laboratory, Milano, Italy). Urinary concentration of nitrite/nitrate was determined by the Griess reagent (Green et al. 1982) (Nitrate/Nitrite colorimetric assay kit; Cayman Chemical, Ann Arbor, MI) using duplicates of urine samples diluted in buffer according to the instruction of the kit. Inulin concentration in urine and plasma samples was determined by the Anthrone method (Fuhr et al. 1955). C_in_ was calculated according to the standard formula. The averaged value of two clearance periods in each experimental phase was used to calculate urine excretory parameters.

### Statistical analysis

For comparisons between the treatment phases versus baseline period in each experimental group repeated measures analysis of variance (ANOVA) was used followed by the Dunnett's posttest, where required. All calculations were done using the GraphPad Prism 5 software (GraphPad Software Inc., La Jolla, CA). *P* = 0.05 was chosen as the significance level. Data are expressed as means ± SEM.

## Results

**Figure 1:**
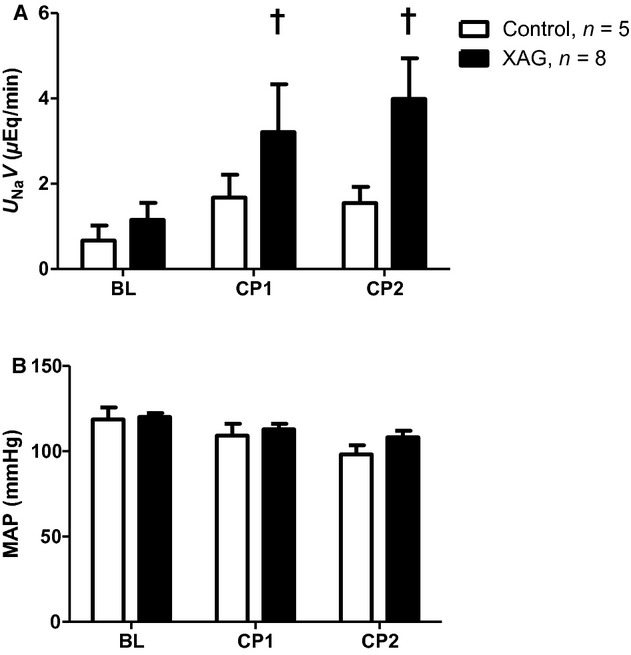
Absolute rate of sodium excretion (A) and mean arterial pressure (B) in control (open bars) and XAG-treated rats. BL, baseline period; CP1, clearance period 1; CP2, clearance period 2. ^†^Statistically significant (*P* < 0.05 or less) compared with baseline period. XAG, 8-*o-β*-d-glucoside derivative of xanthurenic acid.

The renal effects of XAG in normal control rats are depicted in Table 1A and B and in Figure 1. Administration of both the low dose (6.3 nmol) and the high dose (31.5 nmol) of synthetic XAG produced a fourfold increase in urine flow rate compared to baseline values (Table 1A). The diuretic response was accompanied by a mild, dose-related, natriuretic both in absolute rate of sodium excretion (*U*_Na_*V*) (*P* < 0.05, high-dose XAG vs. baseline) and in fractional sodium excretion (%FE_Na_) (*P* < 0.05, high-dose XAG vs. baseline).

**Table 1:** Renal clearance and hemodynamic parameters

	Baseline	Clearance period 1	Clearance period 2
A^1^			
*V* (*μ*L/min)	10.54 ± 2.21	43.91 ± 6.31*	38.63 ± 5.55*
*U*_Na_*V* (*μ*Eq/min)	1.15 ± 0.4	3.21 ± 1.12*	3.99 ± 0.95*
FE_Na_ (%)	0.37 ± 0.12	0.96 ± 0.38	1.63 ± 0.46*
*U*_K_*V* (*μ*Eq/min)	1.34 ± 0.21	2.72 ± 0.19*	2.82 ± 0.19*
FE_K_ (%)	14.32 ± 2.1	22.59 ± 1.6	34.2 ± 2.78*
GFR (mL/min)	1.94 ± 0.19	2.67 ± 0.24	1.94 ± 0.16
MAP (mmHg)	120.1 ± 2.25	112.9 ± 3.31*	108.2 ± 3.85*
B^2^			
*V* (*μ*L/min)	6.44 ± 1.16	13.9 ± 1.78*	7.68 ± 1.6
*U*_Na_*V* (*μ*Eq/min)	0.67 ± 0.36	1.68 ± 0.53	1.55 ± 0.38
FE_Na_ (%)	0.34 ± 0.19	0.49 ± 0.25	0.6 ± 0.26
*U*_K_*V* (*μ*Eq/min)	1.16 ± 0.32	1.49 ± 0.25	1.26 ± 0.26
FE_K_ (%)	14.53 ± 3.9	17.76 ± 4.0	23.95 ± 5.0*
GFR (mL/min)	1.53 ± 0.09	2 ± 0.24	1.39 ± 0.22
MAP (mmHg)	118.7 ± 6.95	109.2 ± 6.84	98.2 ± 5.39*

Data represent the means ± SEM. *V*, urine flow rate; *U*_Na_*V*, absolute rate of sodium excretion; GFR, glomerular filtration rate; FE_Na_, fractional excretion of sodium; FE_K_, fractional excretion of potassium.

* denotes *P* < 0.05.

1 Effects of two incremental doses of XAG on renal clearance parameters in normal rats (*n* = 8).

2 Time control group infused with equivalent volumes of saline instead of the compound (*n* = 5).

The rate of urinary potassium excretion (*U*_K_*V*) also increased significantly with both the low and high doses of the compound (*P* < 0.05). However, the increase in the calculated fractional excretion of K^+^ (FE_K_) did reach statistical significance (*P* < 0.05) only after the high dose of XAG.

The GFR as measured by inulin clearance tended to increase by the low dose of XAG compared with baseline value but this change reached only borderline significance. This may reflect either a true effect of the compound or may be secondary to a non–steady-state condition following the diuresis induced by XAG.

In a time control group (*N* = 5, Table 1B) that was studied similarly but received only vehicle instead of the compound, we observed relatively stable clearance parameters over the entire period of the experiment. There was, however, a slight, yet statistically significant, increase in potassium excretion as well as a significant decrease in MAP (Table 1B).

**Figure 2:**
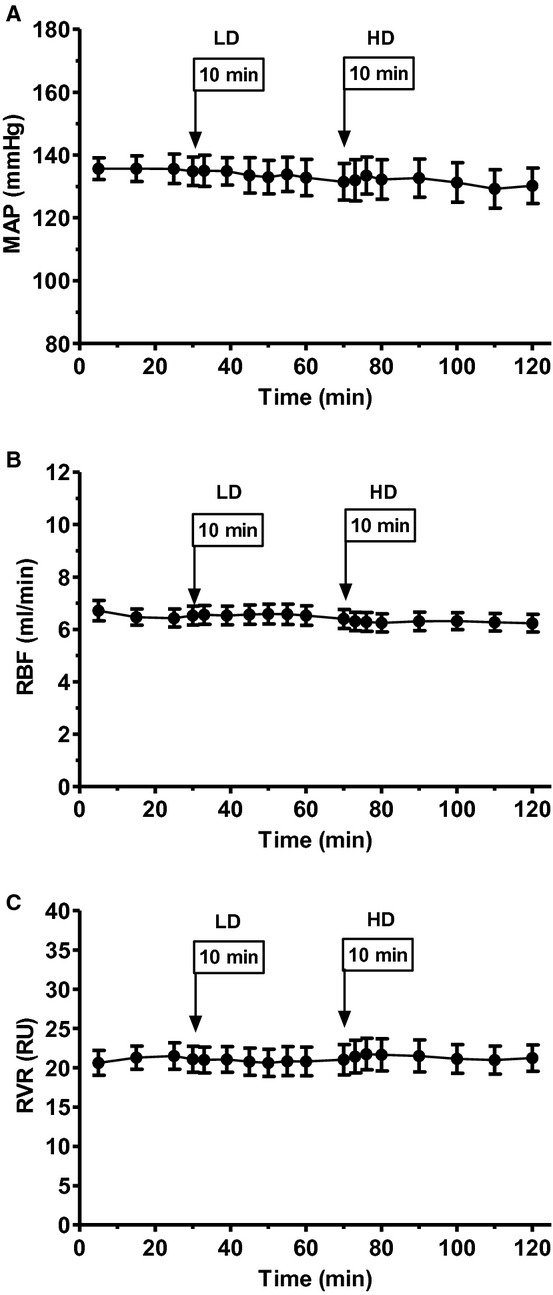
Effects of administration of low dose (LD) and high dose (HD) of XAG on: (A) mean arterial pressure, (B) Renal blood flow, and (C) renal vascular resistance. Data are based on direct measurements by ultrasonic flowmetry (see text for further details). XAG, 8-*o-β*-d-glucoside derivative of xanthurenic acid.

To evaluate whether changes in arterial pressure may be involved in the mechanism of the natriuretic effect of XAG, we compared the changes in MAP and *U*_Na_*V* in the XAG-treated rats with the time control group (Fig. 1). In the time control group, despite a similar decrease in MAP, sodium excretion remained relatively unchanged. This lack of correlation between the changes in MAP and natriuresis suggests that the XAG-induced change in sodium excretion was not related to alterations in MAP. To further clarify whether the renal effects of XAG are hemodynamically mediated, we studied another group of rats with direct measurements of RBF by the ultrasound transit time method, and RVR was continuously calculated and recorded online. These experiments showed that XAG had no significant effect on MAP, RBF, or RVR when administered intravenously both in low or high doses (Fig. 2). Thus, the diuretic and natriuretic actions of XAG appear to be mediated through direct tubular effects.

The data on the experiments with amiloride administration are shown in Table 2. As expected, pretreatment with amiloride in normal rats resulted in significant and sustained diuretic, natriuretic, and antikaluretic effects compared with animals that were not pretreated with amiloride (baseline values in Table 1A and B). In particular, FE_Na_ in the baseline periods of rats pretreated with amiloride (1.3 ± 0.12%) was significantly higher than in the baseline phase of control rats treated with XAG (0.37 ± 0.12), and FE_K_ in amiloride pretreated animals (0.47 ± 0.09) was significantly lower than in control rat treated with XAG (14.32 ± 2.1). This suggests that the amiloride had a significant blocking effect on the epithelial sodium channel in the collecting duct. Administration of XAG on top of amiloride treatment had no further effect on urinary sodium or potassium excretion (Table 2). In fact, in the latter group, there was a slight numerical decrease in sodium excretion that did not reach statistical significance compared with rats treated with amiloride alone.

**Table 2:** Clearance parameters in amiloride pretreated rats

	Baseline		Clearance period 1		Clearance period 2	
			
	−XAG	+XAG	−XAG	+XAG	−XAG	−XAG
*V* (*μ*L/min)	18.64 ± 3.23	17.17 ± 0.99	33.46 ± 5.65^2^	30.24 ± 2.78^2^	35.03 ± 3.82^2^	24.67 ± 3.96
*U*_Na_*V* (*μ*Eq/min)	2.67 ± 0.49	3.02 ± 0.48	4.82 ± 0.92^2^	3.94 ± 0.29	5.29 ± 0.65^2^	3.07 ± 0.33^1^
*U*_K_*V* (*μ*Eq/min)	0.03 ± 0.01	0.04 ± 0.01	0.1 ± 0.03^2^	0.08 ± 0.02^2^	0.11 ± 0.03^2^	0.09 ± 0.02^2^
FE_Na_ (%)	1.78 ± 0.18	1.23 ± 0.12	2.44 ± 0.44	1.83 ± 0.19^2^	2.5 ± 0.41	1.81 ± 0.19^2^
FE_K_ (%)	0.6 ± 0.15	0.62 ± 0.17	1.38 ± 0.54	1.51 ± 0.62^2^	1.49 ± 0.55^2^	1.86 ± 0.61^2^
GFR (mL/min)	1.18 ± 0.17	1.66 ± 0.21	1.58 ± 0.24	1.62 ± 0.17	1.86 ± 0.31^2^	1.32 ± 0.19
MAP (mmHg)	115 ± 4.4	117.3 ± 3.3	111.2 ± 3.1	109.6 ± 3.9^2^	109.9 ± 2.6	103.5 ± 3.3^2^

Effect of amiloride pretreatment on the effects of two incremental doses of XAG on renal clearance parameters in normal rats. Data represent the means ± SEM. *V*, urine flow rate; *U*_Na_*V*, absolute rate of sodium excretion; GFR, glomerular filtration rate; FE_Na_, fractional excretion of sodium; FE_K_, fractional excretion of potassium; *U*_K_*V*, rate of urinary potassium excretion.

1 Statistical significance in −XAG vs. +XAG groups.

2 Statistical significance in Clearance period 1, Clearance period 2 vs. baseline.

**Figure 3:**
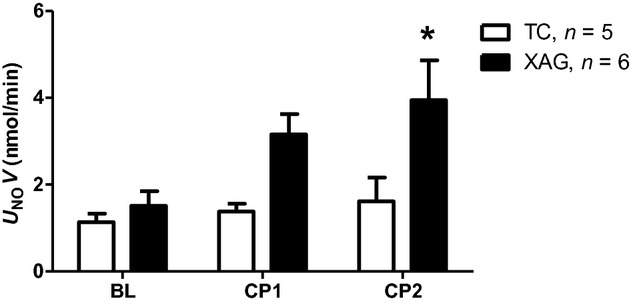
Effect of XAG administration (black bars) versus time control (TC) group (open bars) on urinary excretion of nitrite/nitrate measured by Griess reagent colorimetric assay in clearance studies. BL, baseline period; CP1, clearance period 1; CP2, clearance period 2. *Statistically different compared with baseline value in the same group. XAG, 8-*o-β*-d-glucoside derivative of xanthurenic acid.

**Figure 4:**
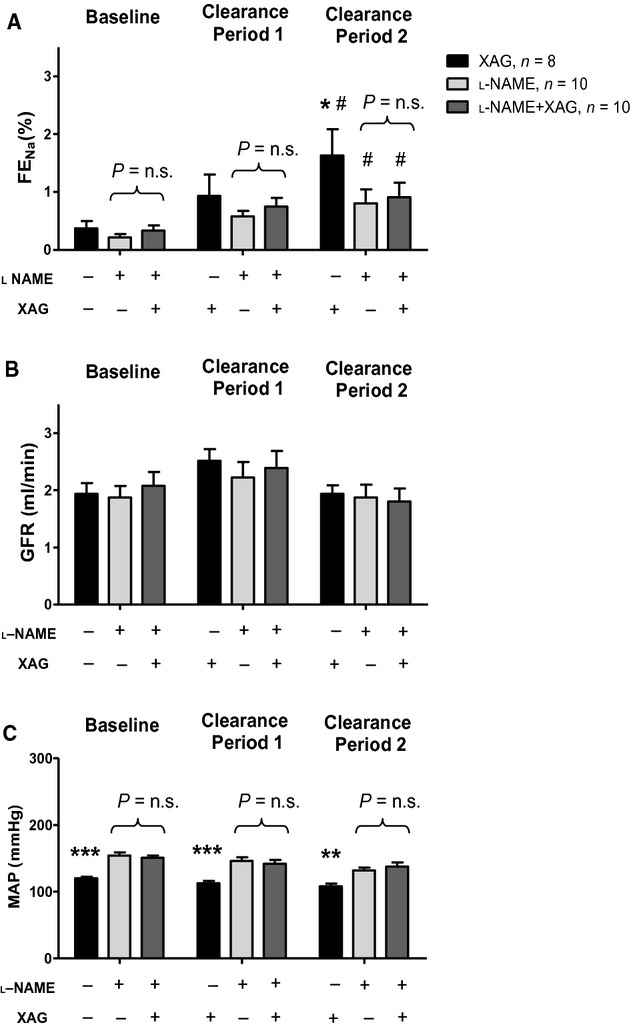
Effects of administration of XAG (black bars), l-NAME (light gray bar), and l-NAME plus XAG (dark gray bars) on (A) fractional sodium excretion, (B) glomerular filtration rate, and (C) mean arterial pressure in clearance experiments described in experimental protocol C (see text for further details). XAG, 8-*o-β*-d-glucoside derivative of xanthurenic acid.

Figure 3 summarizes the data on urinary excretion of nitrite/nitrate in normal rats treated with of XAG compared with time control, vehicle-treated animals. Treatment with two incremental doses of XAG resulted in a dose-related increase (225% and 255%, respectively) in urinary nitrite/nitrate excretion. However, in the time control group, urinary nitrite/nitrate remained unchanged (Fig. 3). Finally, the effect of chronic blockade of the NO system on the renal actions of XAG is summarized in Figure 4. MAP was significantly higher in rats pretreated with l-NAME compared with rats treated with XAG without chronic blockade of the NO system. Administration of XAG to rats pretreated with l-NAME resulted in no further increase in fractional sodium excretion compared with rats with chronic NO blockade not treated with the compound (Fig. 4).

## Discussion

Our results provide confirmation that synthetic XAG has indeed diuretic/natriuretic properties in normal rats. Moreover, our findings suggest that these effects are not mediated by renal hemodynamic changes, but probably are due to a direct tubular action of the compound. Finally, the results demonstrate that the natriuretic/diuretic and kaluretic effects of XAG care completely abolished by pretreatment with amiloride or in the presence of chronic NO blockade. These findings indicate that an intact amiloride-sensitive sodium channel is required for the renal actions of the substance to become manifest, and may suggest that its natriuretic action is mediated in part by a NO-mediated mechanism. Thus, XAG may act in vivo on more than one pathway on different sites along the nephron to promote physiologic diuresis and natriuresis.

Xanthurenic acid is a metabolic product of the essential amino acid tryptophan, via the kynurenine pathway (Schwarcz and Pellicciari 2002; Stone and Darlington 2002). In the brain, tryptophan and its metabolites play an important role in neurotransmission by modulating glutamate receptors activity and possibly nicotinic receptors (Schwarcz and Pellicciari 2002). Xanthurenic acid, as well as other metabolites of tryptophan, is found not only in the brain but also in other organs, including liver and kidney (Allegri et al. 2003). Moreover, several of these tryptophan metabolites are excreted in the mammalian urine (Allegri et al. 1987). In particular, l-kynurenine derivatives were shown to be transported and secreted by the renal organic anion transporters mOAT1 and mOAT3 in the nephron of the murine kidney (Allegri et al. 1987; Bahn et al. 2005). As both XAG and xanthurenic acid sulfate were identified and isolated from urine of patients with chronic renal disease, it is possible that that xanthurenic acid had to become more “hydrophilic” for excretion by the kidney. Most conjugation of hydrophobic compounds for excretion in vertebrates is done with glucuronic acid and not glucose. Alternatively, the conjugation with xanthurenic acid with glucose yields XAG which acts as a hormonal active compound to promote natriuresis and diuresis. This conjugation could be done in the kidney by a member of the UDP glycosyltransferase enzyme superfamily, UGT3A2; an enzyme that is not associated with drug metabolism (Meech et al. 2012a,b).

The effects of tryptophan metabolic products on renal function and, in particular, on the handling of salt and water by the kidney are largely unknown at present. Therefore, the importance of our data stems also from the possibility that xanthurenic acid, and perhaps other tryptophan derivatives, may be a part of a novel endocrine axis involved in the regulation of sodium homeostasis.

Although the mechanism(s) by which XGA promotes sodium and water excretion by the kidney were not elucidated in this study, our data tend to suggest a direct effect on renal tubule rather than a hemodynamically mediated action. Thus, we did not observe significant alterations in RBF in response to i.v. administration of the compound at doses that increased renal salt and water excretion. In addition, the numerical increase in GFR with the low dose of the compound did not reach statistical significance and could be attributed to the brisk diuresis induced by the compound. Admittedly, a significant decrease in MAP was noticed following the administration of the two doses of the compound. Yet, similar changes were observed in the time control group, probably reflecting the effects of prolonged anesthesia (more than 3 h). Indeed, in experiments that were designed to measure the effects of XGA on RBF by direct ultrasonic flowmetry and on MAP, and were of a much shorter duration, we could not detect any effect on either of these parameters. These findings, together with the data presented on the lack of correlation between the changes in MAP and sodium excretion in the clearance studies (Fig. 2), could further attest to a tubular rather than a hemodynamic effect of XAG. Nevertheless, we cannot rule out completely the possibility that subtle changes in glomerular filtration or MAP could contribute to the diuretic/natriuretic actions of the compound.

Our finding of a prompt kaluretic response following the administration of XGA appears to be at odds with those of Cain et al. (2007) who reported only a minimal effect on potassium excretion, suggesting that the compound may act by blocking epithelial sodium channels (ENaC). The reason for the discrepant effects on potassium handling is not clear but may involve differences in dietary potassium intake, ECF volume status, prolonged anesthesia in this study, and in circulating aldosterone levels. In that respect, our findings that amiloride, a blocker of sodium reabsorption in the cortical collecting duct, are of major importance. Our findings clearly demonstrated that pretreatment with amiloride completely abolished both the natriuretic and the kaluretic effects of XAG, suggesting that a functional epithelial sodium channel is mandatory for the renal actions of the compound on sodium and potassium transport. This may be interpreted to support the mechanism proposed Cain et al. (2007), namely, that the main mechanism of action of XAG is by blocking the epithelial sodium channel in the collecting duct. The latter proposal is also compatible with our finding of a lack of an additive natriuretic effect when XAG was given on top of a maximal dose of amiloride. However, if this was the only mechanism of the natriuresis induced by the compound, this could not explain the prompt increase in potassium excretion following XAG administration. Thus, the kaluresis induced by the compound suggests that an additional site of action, that precedes the collecting duct, could be involved in the mechanism of action of XAG. Given the unexpected but consistent effects on potassium excretion, XAG is likely affecting other transporters other than its' effect on ENaC. With respect to the latter option, inhibition of the sodium chloride cotransporter in the early distal tubule or the loop of Henle may also be potential mechanisms for the action of the compound. Nevertheless, we cannot completely rule out the possibility that under normal conditions, in the presence of an unblocked sodium channel, XAG could act also through inhibition of the epithelial sodium channel in the collecting duct.

We have also shown in this study that an intact renal NO system is also necessary for the natriuretic/diuretic action of the compound to become manifest. This is attested by the findings that in the presence of chronic blockade of the NO system administration of the compound did not produce the expected natriuretic/diuretic effect. Most notably is also the finding that administration of XAG in normal rats caused a dose-dependent increase in urinary nitrite/nitrate excretion. These findings suggest that the renal effects of XAG are mediated, at least in part, by activation of the renal NO system. A potential limitation of this interpretation on the effects of XAG on the NO system is the systemic hemodynamic effect of l-NAME as seen in baseline MAP in our study (Fig. 4). However, we think that the hemodynamic effects alone are not sufficient to explain the renal effects of chronic NO inhibition, and other mechanisms are active. In that respect, it should be noted that NO has a modulating inhibitory effect on sodium transport at several sites along the nephron (Garvin et al. 2011). These include the proximal nephron (Guzman et al. 1995), the thick ascending limb of Henle's loop (Ortiz et al. 2001), and the cortical collecting duct (Stoos et al. 1995).

Additionally, in normal man NO mediated natriuresis in response to a sodium load under conditions when NO levels were systemically clamped so not to raise peripheral resistance and thus prevent an increase in MAP (Simonsen et al. 2012). NO clamping induced additional natriuresis in response to volume expansion at constant MABP, GFR, RPF, and decreased renin system activity. Given these constant renal hemodynamic parameters, these results are consistent with the idea that NO acted on the renal tubule as in the present study (Simonsen et al. 2012). It remains to be studied if XAG could be the hormonal signal at the renal tubule to increase natriuresis in response to a sodium load under constant renal hemodynamic parameters as observed in this study.

Admittedly, our study did not identify the site along the nephron, in which sodium transport was inhibited by XGA. Therefore, further studies are required to identify the exact site(s) of action of xanthurenic acid derivatives in the kidney.
